# Exposure to female hormone drugs during pregnancy: effect on malformations and cancer

**DOI:** 10.1038/sj.bjc.6690469

**Published:** 1999-06

**Authors:** E Hemminki, M Gissler, H Toukomaa

**Affiliations:** National Research and Development Centre for Welfare and Health, Health Services Research Unit, PO Box 220, 00531 Helsinki, Finland

**Keywords:** oestrogen, progestin, pregnancy, in-utero, cancer, breast cancer, malformations

## Abstract

This study aimed to investigate whether the use of female sex hormone drugs during pregnancy is a risk factor for subsequent breast and other oestrogen-dependent cancers among mothers and their children and for genital malformations in the children. A retrospective cohort of 2052 hormone-drug exposed mothers, 2038 control mothers and their 4130 infants was collected from maternity centres in Helsinki from 1954 to 1963. Cancer cases were searched for in national registers through record linkage. Exposures were examined by the type of the drug (oestrogen, progestin only) and by timing (early in pregnancy, only late in pregnancy). There were no statistically significant differences between the groups with regard to mothers' cancer, either in total or in specified hormone-dependent cancers. The total number of malformations recorded, as well as malformations of the genitals in male infants, were higher among exposed children. The number of cancers among the offspring was small and none of the differences between groups were statistically significant. The study supports the hypothesis that oestrogen or progestin drug therapy during pregnancy causes malformations among children who were exposed in utero but does not support the hypothesis that it causes cancer later in life in the mother; the power to study cancers in offspring, however, was very low. Non-existence of the risk, negative confounding, weak exposure or low study-power may explain the negative findings. © 1999 Cancer Research Campaign


*British Journal of Cancer* (1999) **80**(7), 1092–1097
© 1999 Cancer Research Campaign
Article no. bjoc.1998.0469

### Exposure to female hormone drugs during pregnancy: effect on malformations and cancer

**E Hemminki, M Gissler and H Toukomaa**

National Research and Development Centre for Welfare and Health, Health Services Research Unit, PO Box 220, 00531 Helsinki, Finland

**Summary** This study aimed to investigate whether the use of female sex hormone drugs during pregnancy is a risk factor for subsequent breast and other oestrogen-dependent cancers among mothers and their children and for genital malformations in the children. A retrospective cohort of 2052 hormone-drug exposed mothers, 2038 control mothers and their 4130 infants was collected from maternity centres in Helsinki from 1954 to 1963. Cancer cases were searched for in national registers through record linkage. Exposures were examined by the type of the drug (oestrogen, progestin only) and by timing (early in pregnancy, only late in pregnancy). There were no statistically significant differences between the groups with regard to mothers' cancer, either in total or in specified hormone-dependent cancers. The total number of malformations recorded, as well as malformations of the genitals in male infants, were higher among exposed children. The number of cancers among the offspring was small and none of the differences between groups were statistically significant. The study supports the hypothesis that oestrogen or progestin drug therapy during pregnancy causes malformations among children who were exposed in utero but does not support the hypothesis that it causes cancer later in life in the mother; the power to study cancers in offspring, however, was very low. Non-existence of the risk, negative confounding, weak exposure or low study-power may explain the negative findings.

**Keywords:** oestrogen; progestin; pregnancy; in-utero; cancer; breast cancer; malformations

Both environmental and endogenous oestrogens have been considered a cause of breast and reproductive-tract cancers (La-Vecchia et al, 1993; McPherson et al, 1996; Leon and Ben-Shlomo, 1997; Møller and Skakkebæk, 1997; Swerdlow et al, 1997*a*; Thomas et al, 1997). It has been further suggested that high levels of endogenous oestrogen in utero increase breast and testis cancer risks in adult life (Ekbom et al, 1992; Michels et al, 1996; Møller and Skakkebæk, 1997; Swerdlow et al, 1997*a*). Hormone drug therapy during pregnancy exposes the mother (at a time of great changes in her body) and the fetus (in a state of organogenesis and rapid growth) to an external source of oestrogens.

Exposure to a synthetic oestrogen, diethylstilbestrol (DES), during pregnancy has been shown to cause anatomical changes in the genitals of both male and female fetuses (Bibbo et al, 1977; Beral and Colwell, 1981; Senekjian et al, 1988) and the exposure is associated with breast cancer of the mother and with benign tumours and cancer of the reproductive tract of daughters (Beral and Colwell, 1980; Colton et al, 1993). For other oestrogen and progestin drugs, some studies show a positive relation to genital malformations, others not (Heinonen et al, 1977; Goldstein et al, 1989; Briggs et al, 1994). In a follow-up study, sons exposed prenatally in utero to medroxyprogesterone had undescended testicles and inguinal hernia more frequently than control sons, but the difference was not statistically significant (Jaffe et al, 1990). Prenatal exposure to oestrogen has been reported to be a risk factor for testicular cancer, cryptorchidism and hypofunctional testes (Depue et al, 1983), and germ-cell tumours of testes and ovaries (Preston-Martin, 1989); there are no previous studies on other cancers of the daughter or any cancer of the mother.

*Received 17 August 1998*
*Revised 30 October 1998*
*Accepted 24 November 1998*

*Correspondence to:* E Hemminki

It is useful to study malformations and cancers at the same time: teratogenic and carcinogenic properties of substances often correlate (Vainio, 1989). For undescended testes and other inborn anatomical changes in the genitals and testicular cancer a common aetiology during embryonic development has been suggested (Møller and Skakkebæk, 1997; Swerdlow et al, 1997*b* ; Prener et al, 1996).

The purpose of this study was to investigate whether the use of female sex hormone drugs during pregnancy is a risk factor for (1) subsequent breast and other oestrogen-dependent cancers among mothers and (2) genital malformations in fetuses. As a by-product of a study investigating the impact of oestrogen and progestin exposure on the fertility of offspring we had follow-up data on the children, and took the opportunity to look at the impact on their risk of cancer, even though the study power for that was very low.

### MATERIALS AND METHODS

This study is based on a retrospective cohort. The information on exposure, confounding factors and immediate outcomes was collected from patient records in maternity centres in Helsinki and that on long-term outcomes was collected from national population and mortality data and from cancer registers through record linkage.

### Cohorts

Since 1944, free prenatal care has been organized by local municipalities. By 1960, 85% of Helsinki women who gave birth had registered in maternity centres. In the 1950s and 1960s, care was given mainly by midwives supported by gynaecologists. A standard maternity care in duplicate was used and one copy was given to the mother. Visits to health care facilities because of pregnancy

**Table 1** Mothers' background characteristics[a]

| (*n*) | Oestrogen[b] Exp (417) | Oestrogen[b] Cont (412) | Progestin only Exp (1566) | Progestin only Cont (1545) | Total[c] Exp (2052) | Total[c] Cont (2038) |
|---|---|---|---|---|---|---|
| Age at first livebirth | | | | | | |
| Mean (s.d.) | 26.3 (5.2) | 25.8 (4.9) | 25.3 (4.8) | 25.0 (4.4) | 25.5 (4.9) | 25.2 (4.5)* |
| ≥ 30, % | 20.4 | 18.0 | 14.5 | 11.7* | 15.8 | 12.9** |
| White collar-worker[d], % | 36.5 | 37.4 | 42.9 | 39.4 | 41.6 | 39.0 |
| Miscarriages prior to index birth, % | 38.9 | 16.3*** | 34.5 | 16.4*** | 35.3 | 16.5*** |
| *Index birth* | | | | | | |
| Pre-pregnancy weight, kg, mean (s.d.) | 57.3 (7.8) | 58.4 (7.6)* | 57.5 (7.9) | 58.2 (8.2)* | 57.5 (7.9) | 58.2 (8.1)** |
| First prenatal visit, wk, mean (s.d.) | 12.3 (4.9) | 17.2 (7.3)*** | 13.2 (5.1) | 16.8 (6.9)*** | 13.1 (5.2) | 17.0 (7.0)*** |
| Other than livebirth, % | 6.9 | 3.0** | 5.3 | 2.7*** | 5.5 | 2.8*** |
| Birthweight[e], g, mean (s.d.) | 3322 (717) | 3477 (572) | 3341 (724) | 3452 (591)*** | 3338 (718) | 3455 (589)*** |
| Not breastfeeding, post-partum visit, % | 21.6 | 23.5 | 25.2 | 26.7 | 24.5 | 25.7 |

[a]Exp = exposed, cont = control group; statistical testing is between exposed and control groups; *$P < 0.05$, **$P < 0.01$, ***$P < 0.001$. Numbers after means in parenthesis give standard deviations. [b]May include also progestin. [c]Includes children with unknown type of exposure. [d]In 1971. [e]If twins, data for the firstborn.

and all drugs prescribed were to be noted on this card, including those prescribed by physicians outside the maternity centre.

A complete list of oestrogen and progestin drugs that were on the Finnish market in the 1950s and early 1960s was made with the help of old drug catalogues. A systematic sample of half (233 of 470) of the boxes containing the maternity cards (in the Helsinki municipality archives) was searched to identify all mothers who had given birth between 1954 and 1963 and who were prescribed these drugs. For each exposed mother the next mother in the file who had given birth during the same year and who was not prescribed hormones was chosen for a control. If the maternity card indicated that the mother had been sent to a hospital for pregnancy-related reasons (18% of the mothers), her records were studied in the hospital archives. If hormone therapy was noted in the hospital record of a control, this control became an exposed mother (1.4% of exposed mothers).

### Malformations

Data on malformations were also extracted from the maternity cards; notes had usually been made by midwives based on the discharge summary sent from the delivery hospital. Data were abstracted and classified by a trained research midwife. Malformations and other problems of the genitals were separately coded, and other malformations were classified into major (such as cleft palate, lacking a limb, or hydrocephalus), and minor (e.g. hip dislocation) and severity unknown.

### Cancers of mothers and children

Follow-up was done with the help of life-long personal identification (ID) numbers given to all Finns between 1964 and 1967. Because our cohort was formed of people born before 1964, ID numbers for the mothers were sought from the Central Population Register, supplemental by data from local church records and death certificates. An ID number was found for 99% of the mothers; the rest had died, emigrated before the ID number was given or lacked an ID number for a reason that remained unclear. For 89% of the index births, a child (children) with an ID number was found. The rest included miscarriages and abortions (7.2%), early deaths (2.9%) and births of unclear outcome (1.5%).

Dates of emigration and death (after the 1964–1967 period) were obtained from the Population Register. Deaths before the 1964–1967 period and causes of death were obtained from the Cause-of-Death Register. Cancers were identified from the National Cancer Register. It has been in operation since 1953 and is based on compulsory notifications by physicians, on data from hospital and laboratory records and on death certificates. The notification rate and accuracy are high (Teppo et al, 1994). The registry used a classification system based on the ICD7 (International Classification of Diseases) for the period 1960–1968 and cancers that occurred before or after this period have been reclassified using the same system of coding.

### Analysis

To study early deaths (stillborn and live born not found in death or population registers) and malformations of the children, all live and stillbirths ($n = 4071$) were included. In studying later deaths and cancer among the offspring, all children with an ID number ($n = 3939$) were included. For the study of maternal cancer; all mothers with an ID number were included ($n = 4090$).

The original pairs of exposed and control mothers were used to form the following groups and their control groups: mothers and children exposed to (1) oestrogen drugs (with or without progestin drugs) (at least) early in pregnancy, (2) oestrogen drugs (with or without progestin drugs) only late or at an unknown time, (3) just progestin drugs (at least) early in pregnancy, (4) just progestin drugs only late in pregnancy or at an unknown time. Those exposed to hormone drugs the content of which could not be identified, together with their controls, were included in the total figures. Early exposure was defined to include a prescription or an injection in the first 16 gestation weeks calculated from the last menstrual period, or if that date was lacking, when the indication for the drug was either (recurrent) miscarriage or nausea. Individual mothers could have received more than one drug and, in defining the exposure group, drugs from various sources (maternity centre, hospital) at different times were combined. Due to the sampling method, one mother could appear more than once (6.4% of all mothers), and her group could be different for different index births. In the analysis of maternal cancer, each mother was counted only once, and her exposure group was chosen in the order of the groups given above.

© 1999 Cancer Research Campaign

**Table 2** Follow-up on mothers until 1997[a], cancers after the index birth (in 1954–1963)

| (Number of mothers) | Oestrogen[b] Exp (417) | Oestrogen[b] Cont (412) | Progestin only Exp (1566) | Progestin only Cont (1545) | Total[c] Exp (2052) | Total[c] Cont (2038) |
|---|---|---|---|---|---|---|
| All cancers[e] | | | | | | |
| Incidence (*n*) | 34.7 (49) | 39.6 (56) | 41.7 (221) | 41.4 (212) | 40.9 (284) | 40.9 (278) |
| OR (CI) | 0.87 (0.59–1.28) | | 1.01 (0.84–1.22) | | 1.00 (0.85–1.18) | |
| Adj. OR (CI) | 0.85 (0.56–1.28) | | 1.02 (0.83–1.25) | | 1.00 (0.83–1.20) | |
| Breast cancer | | | | | | |
| Incidence (*n*) | 12.0 (17) | 12.7 (18) | 13.4 (71) | 14.5 (74) | 13.7 (95) | 14.0 (95) |
| OR (CI) | 0.95 (0.49–1.83) | | 0.93 (0.67–1.29) | | 0.98 (0.74–1.30) | |
| Adj. OR (CI) | 0.96 (0.49–1.90) | | 0.94 (0.69–1.32) | | 1.00 (0.74–1.34) | |
| Ovarian cancer | | | | | | |
| Incidence (*n*) | 2.1 (3) | 0.7 (1) | 1.3 (7) | 2.3 (12) | 1.4 (10) | 2.2 (15) |
| OR (CI) | 3.00 (0.31–28.9) | | 0.56 (0.22–1.43) | | 0.65 (0.29–1.45) | |
| Adj. OR (CI) | 3.28 (0.34–32.0) | | 0.69 (0.27–1.79) | | 0.73 (0.32–1.63) | |
| Cervical cancer[d] | | | | | | |
| Incidence (*n*) | 5.0 (7) | 3.5 (5) | 2.6 (14) | 3.9 (20) | 3.0 (21) | 3.7 (25) |
| OR (CI) | 1.40 (0.44–4.41) | | 0.68 (0.34–1.34) | | 0.82 (0.46–1.47) | |
| Adj. OR (CI) | 1.51 (0.47–4.83) | | 0.61 (0.30–1.24) | | 0.83 (0.46–1.50) | |
| Uterine cancer | | | | | | |
| Incidence (*n*) | 1.4 (2) | 0 (0) | 2.6 (14) | 1.4 (7) | 2.3 (16) | 1.0 (7) |
| OR (CI) | – | | 1.94 (0.78–4.79) | | 2.24 (0.92–5.44) | |
| Adj. OR (CI) | – | | 1.67 (0.66–4.23) | | 1.98 (0.80–4.87) | |

[a]Exp = exposed, cont = control group, OR = odds ratio, CI = 95% confidence limits, Adj. = adjusted for age at first livebirth, number of live births, pre-pregnancy weight. Incidence is per 10 000 person-years; follow-up until 1997, emigration or death (mean 35 years). In logistic regression 'cont' is the reference group. [b]May include also progestin. [c]Includes children with unknown type of exposure. [d]With cancer in situ. [e]Each mother only once.

For the estimations of incidence, the number of cancers was divided by person-years calculated from the date of the index birth (in the case of mothers who appeared more than once, from the index birth defining her exposure group) to March 1997, emigration or death, whichever was first. Cancers occurring prior to the index birth were ignored. $\chi^2$-tests and *t*-tests were used to assess the statistical significance of the differences. Odds ratios (ORs) and their 95% confidence intervals (CIs) were calculated for cancer incidence. Differences between the groups were adjusted by logistic regression for the continuous variables: 'age at first live birth', 'number of live births', 'pre-pregnancy weight' as confounders. In a second analysis, 'miscarriages prior to the index birth' (continuous) was added, and in a third, 'current pregnancy ending in miscarriage' (yes-no) was further added.

### RESULTS

Of the 100 different drug trade-names containing oestrogen and progestins that were on the market, 32 had been used. The most common drug was Primolut® (37% of the drugs mentioned). It contained either progesterone, oxyprogesterone, ethisterone, or norethisterone (depending on the drug form). The next most common ones were Progesteron® (either progesteron or ethisterone) (26%), Gestanon® (allylesterone) (12%) and Di-Pro® (a combination of estradiol or ethinylestradiol and progesterone or ethisterone) (9%). There was no case of DES-exposure. The most common indications for hormone drugs were threatened miscarriage (47%, counted from the first drug exposure), recurrent miscarriage (9%), and threatened premature birth (7%). The length of the therapy could be calculated for only half of the women, and for 68% of these the exposure had been for less than 4 weeks. However, most (77% of all drugs mentioned) were administered by injection and in the case of debot (slow-releasing) forms the influence could have lasted for weeks.

In most respects the exposed and control mothers were similar (Table 1). Some differences were statistically significant but, in absolute terms, the differences were small. However, previous to the index birth, the exposed mothers had more often had pregnancies not ending in a live birth. Their index pregnancies, by definition, were more problematic than those of the control mothers, and the children were more often of low birth-weight.

### Mothers

During the follow-up period (on average 35 years) the number of exposed and control mothers who developed cancer was equal (Table 2). When mothers who had only basal cell carcinoma of the skin, in situ cervical cancer and other 'benign' cancers were excluded, the similarity between the groups remained.

Numbers were largest for cancer of the breast, and for this site the ORs were particularly evenly distributed between the exposed and control women (Table 2). When examined by a more detailed grouping, there was an indication that mothers exposed to oestrogen in early pregnancy developed breast cancer more frequently (OR = 1.30, CI 0.57–2.97) and mothers exposed to oestrogen in late pregnancy less frequently (OR = 0.49, CI 0.15–1.62), but the numbers were small and the confidence intervals large. Timing of progestin exposure did not have any influence on risk. Adjustment for confounding factors did not change the results (Table 2).

Ovarian and cervical cancer were more common among oestrogen-exposed mothers and less common among progestin-

 *© 1999 Cancer Research Campaign*

**Table 3** Numbers of malformed children by exposure group[a]

| | Oestrogen[b] | | Progestin only | | Total[c] | |
|---|---|---|---|---|---|---|
| | Exp | Cont | Exp | Cont | Exp | Cont |
| (Denominator) | (409) | (425) | (1484) | (1601) | (1963) | (2108) |
| Male genital | 3 | 0 | 3 | 0 | 6 | 0** |
| 'Related'[d] | 0 | 2 | 3 | 4 | 3 | 6 |
| Other major | 4 | 0* | 15 | 8 | 19 | 9* |
| Other minor | 2 | 2 | 31 | 17* | 35 | 21* |
| Other, severity unknown | 5 | 2 | 8 | 2* | 13 | 4* |
| Total | 14 | 6 | 60 | 31*** | 76 | 40*** |

[a]Statistical testing is between exposed and control groups, *$P < 0.05$, **$P < 0.01$, ***$P < 0.001$. [b]May contain also progestin. [c]Includes children with unknown type of exposure. [d]The group 'related' includes children with swollen breasts (2 exposed and 2 controls), microanus (1 exposed), bleeding from the vagina and phimosis (both controls), and one unclear diagnosis, apparently a perianal fistula (1 control).

**Table 4** Follow-up of children until the age of 34–43-years[a]

| | Oestrogen[b] | | Progestin only | | Total[c] | |
|---|---|---|---|---|---|---|
| | Exp | Cont | Exp | Cont | Exp | Cont |
| Children followed, *n* | (389) | (414) | (1432) | (1557) | (1890) | (2049) |
| Cancer cases | | | | | | |
| Men | 2 | 1 | 7 | 5 | 9 | 7 |
| Women | 2 | 4 | 8 | 12 | 10 | 16 |
| Cancer incidence[d] | | | | | | |
| Men | 2.8 | 1.5 | 2.8 | 1.8 | 2.7 | 2.0 |
| Women | 3.0 | 5.3 | 3.3 | 4.7 | 3.1 | 4.6 |
| Odds ratios (95% CI)[e] | | | | | | |
| Men | 1.95 (0.17–21.5) | | 1.58 (0.50–4.97) | | 1.40 (0.52–3.76) | |
| Women | 0.57 (0.11–3.13) | | 0.69 (0.28–1.70) | | 0.67 (0.30–1.47) | |

[a]Statistical testing is between exposed and control groups, *$P < 0.05$, **$P < 0.01$, ***$P < 0.001$. [b]May contain also progestin. [c]Includes children with unknown type of exposure. [d]Per 10 000 person-years; follow-up until 1997, emigration or death (mean 36 years). [e]Control = 1, CI = confidence interval.

exposed mothers (Table 2), but the differences were not statistically significant. When the cases of cancer in situ were excluded, the relative differences remained the same.

Uterine cancer was more common among the exposed mothers, even when examined in more detail by timing of exposure. The difference was not statistically significant, but was more suggestive of a genuine elevation of risk than any of the results for the other genital cancers.

Further adjustment for previous miscarriages or miscarriages in the index births did not notably change the adjusted odds ratios for cancers shown in Table 2.

Colon cancer was more common among the exposed mothers, but not statistically significantly so (OR = 1.59, CI = 0.66–3.85). There was more lung cancer among mothers who had received progestin drugs in early pregnancy (OR = 10.5, CI = 1.36–81.4).

According to the Cause-of-Death Register there were 65 cancer deaths among the exposed mothers and 77 among the controls. The difference was due to more breast cancer deaths among the control ($n = 20$) than exposed mothers ($n = 11$, $P = 0.053$). Other reproductive cancer deaths were rarer and relatively evenly distributed between the exposed and control mothers.

### Children

The total number of malformations, as well as the number of male genital problems recorded on the maternity cards, was higher among the exposed children (Table 3). The genital problems were: hydrocele (two boys), hypospadia (two), operation on the scrotum (one for haematoma, one for necrotic testis). With the exception of one child, mothers of these boys had been given oestrogen- and/or progestin-containing intramuscular injections in early pregnancy; the number of recorded injections varying from 2 to 11. The mother of one child had had progestin injections 3 months before delivery.

There were more early deaths in the exposed groups, and fewer in later life. However, the differences were not significant. In the follow-up period (mean 36 years), incidence of cancer and the ORs were somewhat higher among exposed men than control men, but the opposite was found for women. Again, however, the numbers were small and all differences were statistically non-significant. Among men, no cancers of the male genitalia occurred. Among women, there were seven cases of breast cancer (three exposed, four controls), one of ovarian cancer (control), and three of cancer of the uterine cervix (one exposed, two controls). There were no cases of clear cell adenocarcinoma or cancer in situ of the cervix uteri. Cancer as the main cause of death was recorded for four exposed and one control child.

### DISCUSSION

Our study did not support the hypothesis that oestrogen or progestin drug therapy during pregnancy causes cancer in the mother

© 1999 Cancer Research Campaign or in offspring later in life. However, it could be that confounding by unmeasured factors and weak exposure contribute to the observed results. The exposed mothers, by definition, had had more problems during the index pregnancy, but these also occurred in prior pregnancies (as judged by the number of miscarriages). Most hormones were prescribed for indications such as miscarriage or preterm birth or the threat of them, and the birthweights of the children of the index births were lower. If these indicate lower endogenous oestrogen production during pregnancy (Ekbom et al, 1992; Michels et al, 1996), then low endogenous oestrogen production may be a negative confounder in our study masking any impact the hormone drugs may have had. Furthermore, negative confounding could have been caused by other differing characteristics not recorded in our data sources. The finding that there were more lung cancers among progestin-exposed mothers may indicate that more of these mothers smoked or that they smoked more. Smoking could have caused the pregnancy problem (Walsh, 1994) and decreased the incidence of hormone-dependent cancers, such as uterine cancer (Wald and Hackshaw, 1996).

Details of the timing and length of therapy were not always given, but some of the therapies were short. If there is a threshold, with dosages only effective above a certain minimum, or if there is a very narrow time-window during gestation when a patient is vulnerable, then many of the women were not truly exposed. Furthermore, a great variety of different types of oestrogen and progestins were prescribed, and even minor modifications in the molecular structure of a drug formulation can substantially effect the biological properties.

For the study of cancer among the offspring of the exposed women the numbers were very small but, nonetheless, gave no indication of a large increase in risk. After 10 or 20 years, when the subjects are in an age-group that is more affected by cancer, it will be worth studying the children anew. There were more malformations among exposed children. This suggests that oestrogen and progestin exposure could be a risk-factor for subsequent cancers that would be detected only by longer follow-up or in a larger study. On the other hand, causality may have been the other way around: a malformation in the fetus may have caused symptoms resulting in the hormone therapy.

It is notoriously difficult to study drug effects occurring 40 years after their use in a scenario of rapidly changing clinical practice and with a large number of pharmacological modifications. Confounding by indications for treatment is an intrinsic problem that can only be properly resolved in a clinical trial. To our knowledge no such trial with progestins or oestrogen other than DES have been carried out, and these trials are unlikely to be undertaken in view of current understanding of the ineffectiveness of the therapy (Goldstein et al, 1989).

The importance of long-term follow-up studies on hormone drug therapy during pregnancy can be argued on two grounds. First, even though treating pregnancy problems with oestrogen drugs is no longer popular, the treatment of threatened miscarriage with progestin and some other hormones still occurs. Furthermore, women are exposed intentionally and unintentionally to hormone drugs, either during pregnancy or immediately before it. In the latter event, if the half-life of the drug is long it may still be in the body during pregnancy. Such exposures may result from failed long-term intradermal or injection contraceptives, postcoital contraceptive pills, treatment of (assumed) premenstrual syndrome, assisted conception techniques (IVF and others) and oestrogen-containing natural products. Secondly, studying effects on cancer may be educational in illustrating how important it is to make a full evaluation of the outcomes of a drug therapy during pregnancy, including long-term effects. Such illustration may contribute to better criteria for defining the safety of drugs. Acknowledging the possibility of long-term effects is also an argument for taking a conservative approach in treating pregnant women with drugs, and also for use of a small number of drugs instead of many different modifications, to facilitate more complete evaluation.

### ACKNOWLEDGEMENTS

We thank Erja Forssas, Jouni Meriläinen and Eija Vuori for their help in the data collection and data analysis, Eero Pukkala and the Finnish Cancer Register for cancer data, and Jaakko Tuomilehto for comments.

### REFERENCES

Beral V and Colwell L (1980) Randomized trial of high doses of stilbestrol and ethisterone in pregnancy: long-term follow-up of mothers. *Br Med J* **281**: 1098–1101

Beral V and Golwell L (1981) Randomized trial of high doses of stilboestrol and ethisterone therapy in pregnancy: long-term follow-up of the children. *J Epidemiol Commun Health* **35**: 155–160

Bibbo M, Gill WM, Azizi F, Blough R, Fang VS, Rosenfield RL, Schumaher GFB, Sleeper K, Sonek MG and Wied GL (1977) Follow-up study of male and female offspring of DES-exposed mothers. *Obstet Gynecol* **49**: 1–8

Briggs GG, Freeman RK and Yaffe SJ (1994) *Drugs in Pregnancy and Lactation*, 4th Edn, pp. xi, xii. Williams & Wilkins: Baltimore

Colton T, Greenberg R, Noller K, Resseguie L, Van Bennekom C, Heeren T and Zhang Y (1993) Breast cancer in mothers prescribed stilbestrol in pregnancy: further follow-up. *JAMA* **269**: 2096–2100

Depue RH, Pike MC and Henderson BE (1983) Estrogen exposure during gestation and risk of testicular cancer. *J Natl Cancer Inst* **71**: 1151–1155

Ekbom A, Trichopoulos D, Adami H-O, Hsieh C-C and Lan S-J (1992) Evidence of prenatal influences on breast cancer risk. *Lancet* **340**: 1015–1018

Goldstein PA, Sacks HS and Chalmers TC (1989) Hormone administration for the maintenance of pregnancy. In *Effective Care in Pregnancy and Childbirth*, Chalmers I, Enkin M, Keirse MJNC (eds), pp. 612–623. Oxford University Press: Oxford

Heinonen OP, Slone D and Shapiro S (1977) *Birth Defects and Drugs in Pregnancy*. Publishing Sciences Group: Littleton, Mass

Jaffe B, Shye D, Harlap S, Baras M, Belmaker E, Gordon L, Magidor S and Fortney J (1990) Health, growth and sexual development of teenagers exposed in utero to medroxyprogesterone acetate. *Pediatric Perinatal Epidemiol* **4**: 184–195

La-Vecchia C, Negri E, Franceschi S and Parazzini F (1993) Long-term impact of reproductive factors on cancer risk. *Int J Cancer* **53**: 215–219

Leon D and Ben-Shlomo Y (1997) Preadult influences on cardiovascular disease and cancer. In *A Life Course Approach to Chronic Disease Epidemiology*, Kuh D, Ben-Shlomo Y (eds). Oxford University Press: Oxford

McPherson CP, Sellers TA, Potter JD, Bostick RM and Fosom AR (1996) Reproductive factors and risk of endometrial cancer. The Iowa Women's Health Study. *Am J Epidemiol* **143**: 1195–1202

Michels KB, Trichopoulos D, Robins JM, Rosner BA, Manson JE, Hunter DJ, Colditz GA, Hankinson SE, Speizer FE and Willett WC (1996) Birthweight as a risk factor for breast cancer. *Lancet* **348**: 1542–1546

Møller H and Skakkebæk NE (1997) Testicular cancer and cryptorchidism in relation to prenatal factors: case-control studies in Denmark. *Cancer Causes Control* **8**: 904–912

Prener A, Engholm G and Jensen OM (1996) Genital anomalies and risk for testicular cancer in Danish men. *Epidemiology* **7**: 14–19

Preston-Martin S (1989) Epidemiological studies of perinatal carcinogenesis. In *Perinatal and Multigeneration Carcinogenesis*, Napalkov NP, Rice JM, Tomatis L, Yamasaki H (eds), pp. 289–334. IARC Scientific Publications No 96. IARC: Lyon

Senekjian EK, Potkul RK, Frey K and Herbst AL (1988) Infertility among daughters either exposed or not exposed to diethylstilbestrol. *Am J Obstet Gynecol* **158**: 493–498

© 1999 Cancer Research Campaign

Swerdlow AJ, De Stavola BL, Swanwick MA and Maconochie NES (1997*a*) Risks of breast and testicular cancers in young adult twins in England and Wales: evidence on prenatal and genetic aetiology. *Lancet* **350**: 1723–1728
Swerdlow AJ, Higgins CD and Pike MC (1997*b*) Risk of testicular cancer in cohort of boys with cryptorchidism. *Br Med J* **314**: 1507–1511
Teppo L, Pukkala E and Lehtonen M (1994) Data quality and quality control of a population-based cancer registry. Experience in Finland. *Acta Oncol* **33**: 365–369
Thomas HV, Reeves GK and Key TJA (1997) Endogenous estrogen and postmenopausal breast cancer: a quantitative review. *Cancer Causes Control* **8**: 922–928
Vainio H (1989) Carcinogenesis and teratogenesis may have common mechanisms. *Scand J Work Environ Health* **15**: 13–17
Wald NJ and Hackshaw AK (1996) Cigarette smoking: an epidemiological overview: *Br Med Bull* **52**: 3–11
Walsh RA (1994) Effects of maternal smoking on adverse pregnancy outcomes: examination of the criteria of causation. *Hum Biol* **66**: 1059–1092

© 1999 Cancer Research Campaign
